# Filling Frail Teeth with Gold

**Published:** 1874-12

**Authors:** H. L. Ambler


### FILLING FRAIL TEETH WITH GOLD.

BY H. L. AMBLER, D. D. S.

There is a certain class of teeth which is second or third rate in structure, being designated as chalky, and of an opaque white or brownish white color. Filling these teeth with gold so as to preserve them for years is our subject, but more especially do we speak of proximal cavities in these teeth; however, the same general principles will apply to cavities in any surface of the same. We are convinced that failures have been made upon such frail teeth, by trying to produce full contours, in filling with extra cohesive foil, highly annealed. For in malleting the gold against the margins they are bruised to such an extent, that in a short time decay begins again, Before excavating, we would make a free V shaped separation, for the superior teeth, with the open portion of the V toward the palatine arch, and for the inferior toward the tongue. This will often give such an amount of room, that wedging to obtain space will be unnecessary; simply use in such cases, a piece of compressed cotton-wood over the rubber-dam, below the base of the cavity, dampening the inside end of the wood so that it will not slip or come out; this serves as a guard against the slipping of instruments and helps to hold the teeth firm. In many cases where the base of the cavity is far below the margin of the gum, and there is scarcely room for a wooden or metal shield, in order to keep wedges from slipping on the rubber-dam, we would use a floss silk ligature tied around the necks of the two adjoining teeth, and as a part of the floss touches the wood, it will keep it firmly in place. In excavating never stop until arriving at the extreme borders of disintegration, as it is preferable to have the cavities as wide as the tooth will admit from buccal to palatine or lingual, as the case may be; for if after the teeth are filled, and they should change position at all, in this case the gold surfaces would come in contact; whereas, if the cavities were narrow the tooth substance might again touch. Be sure that the margins are as smooth as they can be made before beginning to fill; a piece of fine sand or emery paper will here be of use, and if the cavities are anything but small begin at the masticating surface to excavate. For filling, we would use Williams' No. 30 rolled gold, slightly annealed by passing the strips once through the flame of the spirit lamp. This gold is soft, pliable, easy to manipulate and not liable to clog, as some of the more adhesive and stiff foils; it will make a filling as hard as such teeth will bear, and it is more apt to be impervious to moisture and remain so for a longer time. For driving the gold to the margins, we would use a flat-faced, smooth instrument like No. 5 in Dr Butler's new set, always being sure to keep the gold between the instrument and the tooth, in this manner, you are not liable to fracture or crush the margins, which are among the main things to be guarded against; then let the gold be built out slightly convex, to prevent any future contact of the teeth. In saving this class of teeth much care is required, also some conservation; therefore, should there be any points which you can not carefully reach with your mallet instrument, let hand force be employed, finally going over the whole surface of the filling with a burnisher after the gold is all packed and also after it is finished. Sometimes a small portion of gold will lap over a frail wall of the cavity, then if you should use your mallet burnisher to excess, in going over this everlapping piece, you are liable to cause a fracture, thus jeoparding the whole operation. We will find some of these cases where only the walls of enamel are left, but we think they can be made practically useful by the above method.

